# Quality of life amongst adolescents and young adults with cystic fibrosis: correlations with clinical outcomes

**DOI:** 10.6061/clinics/2017/e427

**Published:** 2018-10-05

**Authors:** Daniela W Gancz, Maristela T Cunha, Claudio Leone, Joaquim C Rodrigues, Fabíola V Adde

**Affiliations:** I Unidade de Pneumologia Pediatrica, Instituto da Crianca, Hospital das Clinicas HCFMUSP, Faculdade de Medicina, Universidade de São Paulo, Sao Paulo, SP, BR; II Servico de Fisioterapia, Instituto da Crianca, Hospital das Clinicas HCFMUSP, Faculdade de Medicina, Universidade de Sao Paulo, Sao Paulo, SP, BR; III Departamento de Saude Materno Infantil, Faculdade de Saude Publica, Universidade de Sao Paulo, Sao Paulo, SP, BR

**Keywords:** Cystic Fibrosis, Quality of Life, Questionnaires, Adolescence

## Abstract

**OBJECTIVES:**

The current study sought to evaluate the quality of life of young patients with cystic fibrosis and correlate these results with the clinical parameters indicative of disease severity.

**METHODS:**

This cross-sectional study applied the validated Portuguese version of a cystic fibrosis specific quality of life questionnaire to clinically stable patients aged 14 to 21 years old. The correlations between the questionnaire domain scores and forced expiratory volume in one second (FEV_1_) values, the Shwachman-Kulczycki score, and body mass index were assessed, and correlations were considered as significant when *p*<0.05.

**RESULTS:**

A total of 31 patients (11 females; 16.4±2.3 years old) were evaluated, and the median scores on the questionnaire domains ranged from 66.7 to 100. A significant correlation was found between body mass index and the weight (r=0.43, *p*=0.016) and the eating questionnaire domains (r=0.44, *p*=0.013); between FEV_1_ and the physical (r=0.53, *p*=0.002) and treatment burden (r=0.41, *p*=0.023) domains; and between the Shwachman-Kulczycki score and the physical (r=0.39, *p*=0.03), health (r=0.41, *p*=0.023), and role (r=0.37, *p*=0.041) domains. A significant difference was found amongst patients with FEV_1_ values above or below 60% of the predicted value with regard to the role and health domains. No differences in the scores were found according to gender.

**CONCLUSIONS:**

The current cystic fibrosis specific quality of life questionnaire scores exhibited wide variability across all domains; however, they indicated a relatively satisfactory quality of life amongst the patients studied. Certain domains exhibited significant correlations with clinical parameters; thus, this instrument has consistent associations with clinical outcomes.

## INTRODUCTION

The survival of patients with cystic fibrosis (CF) has increased significantly over recent decades because of earlier diagnoses, better approaches to the treatment of lung and pancreatic diseases, and improvements in nutrition. Half of the current CF population in developed countries consists of adults 1,2. The symptoms of CF and their complex treatment regimen impose a high burden on both patients and parents, eventually increasing their levels of stress and affecting their quality of life 3,4.

Health-related quality of life (HRQoL) measures and assesses symptoms and daily functioning from the patient's perspective. Quality of life (QoL) measurements have been performed amongst patients with CF with increasing frequency over recent years to assess the physical, social, and psychological effects of this disease as well as to evaluate therapeutic interventions 5-7. These data provide clinicians with valuable disease-specific information across multiple functional dimensions.

The Cystic Fibrosis Questionnaire-Revised (CFQ-R) 8-11 is a CF–specific instrument that is valuable for obtaining patient-reported assessments of various health-related measures. A validated Portuguese version of this scale exists and shows satisfactory reproducibility across all of its domains 12.

Four versions of the CFQ-R exist: for ages 6 to 11 (interview) evaluating 8 domains; for ages 12 to 13 (self-report) evaluating 8 domains; a parent version for children between 6 and 13 years evaluating 11 domains; and a teen/adult version (self-report) for ages 14 years through adulthood 10. The teen/adult version assesses 12 domains: physical functioning, role, vitality, emotional functioning, social, body image, eating disturbances, treatment burden, health perceptions, weight, respiratory symptoms, and digestive symptoms. Each domain is composed of a variable number of self-report questions with four possible answers, with a total of 50 questions. The scores range from 0 to 100, with higher scores indicating a higher patient-reported quality of life with regard to the domain being evaluated. The CFQ-R has a manual and a computerised version to calculate the scores that are available upon request 11.

Despite the increasing use of the CFQ-R by several studies, consensus does not yet exist regarding the interpretation of its scores within the clinical context 5,13. In addition, uncertainty exists concerning the association between CFQ-R scores and clinical outcomes such as pulmonary function, chest computed tomography scores, nutritional status, and exacerbations 9,14-17. The present study sought to assess CFQ-R scores in a population of adolescents and young adults with CF and evaluate the correlations between the scores of specific domains and the clinical and laboratory features of the disease.

## MATERIALS AND METHODS

This cross-sectional study examined patients with CF followed at the pulmonology outpatient clinics of the Children's Institute or the Central Institute of the Hospital das Clínicas, University of São Paulo. This study was approved by the Human Ethics Committee of the Hospital das Clínicas of the Medical School of the University of São Paulo (Faculdade de Medicina da Universidade de São Paulo; FMUSP; approval No. 0345/09). The study participants or their legal guardians signed an informed consent form. The inclusion criteria were a confirmed diagnosis of CF according to the Cystic Fibrosis Foundation guidelines 18, an age between 14 to 21 years, and clinical stability over the previous 15 days. Patients submitted to lung transplantation were excluded.

Following their agreement to participate, the patients completed the CFQ-R teen/adult version during a routine visit to the outpatient clinic. The questionnaire was always administered by the same physician.

The demographic and clinical characteristic data of the participants collected from their clinical records were as follows: presence of pancreatic insufficiency; bronchial colonisation; and routine treatments. Body mass index (BMI), forced expiratory volume in one second (FEV_1_), and the Shwachman-Kulczycki score (SKS) 19 were obtained during the same clinic visit when the CFQ-R was completed. Pancreatic insufficiency was diagnosed clinically, inferred by the presence of frequent, greasy stools and a qualitative microscopic evaluation of faecal fats (Sudan III stain). A sputum culture was used to monitor bronchial colonisation. If the patient was not able to expectorate, then an oropharyngeal swab was collected. Leeds criteria were used to classify chronic infection 20. Spirometry was performed following the recommendations of the American Thoracic Society/European Respiratory Society (ATS/ERS) 21 at the pulmonary function laboratory of the Instituto da Criança. Reference equations were selected according to age 22,23, and the ATS criteria were used to classify the severity of the respiratory disorder 24.

The scores of each CFQ-R domain were calculated, and the following correlations were assessed: BMI versus the body image, weight, and eating domains; FEV_1_ versus the physical, respiratory, treatment burden, and vitality domains; and the SKS versus the vitality, physical, health, social, role, and emotional domains.

The scores for each CFQ-R domain were compared between groups categorised based on gender and FEV_1_. The criterion selected (FEV_1_ above or below 60% of the predicted value) was based on the following ATS/ERS standards for interpreting spirometry results: FEV_1_ between 60-79% of the predicted value: mild-to-moderate obstruction; FEV_1_<60%: moderately severe, severe, or very severe obstruction 24.

The time needed to complete the CFQ-R was recorded.

### Statistical analyses

A convenience sample of 31 patients who fulfilled the inclusion criteria was retrieved from the CF follow-up clinic at our institution. It was estimated *a posteriori* that a sample size of 29 would have 80% power to detect a correlation coefficient of at least 0.5 with an alpha of 5% (MedCalc^®^ software version 12.0.0.0) between FEV_1_ and the physical functioning domain.

A descriptive analysis of the demographic and clinical characteristics of the sample was performed, and the results were expressed as the number of cases, mean ± standard deviation, or median.

The correlations were assessed using Spearman's correlation coefficient and a linear regression.

The scores in each CFQ-R domain were compared as a function of gender and FEV_1_ using the unpaired Student's t-test or the Mann-Whitney test. When the standard deviation between groups was large, Welch's correction was used to assess the normality of the data distribution. These calculations were performed using Graph Pad InStat software.

## RESULTS

This study was conducted from June 2009 to April 2012. The population eligible to participate in the study corresponded initially to 60 individuals; 29 patients were not included because of respiratory instability or no wish to participate. The demographic and clinical characteristics of the 31 patients included are described in Table 1.

The patients completed the CFQ-R in 10 to 30 minutes (median=14 minutes). The scores of all the domains are displayed in Table 2.

**Figure 1:**
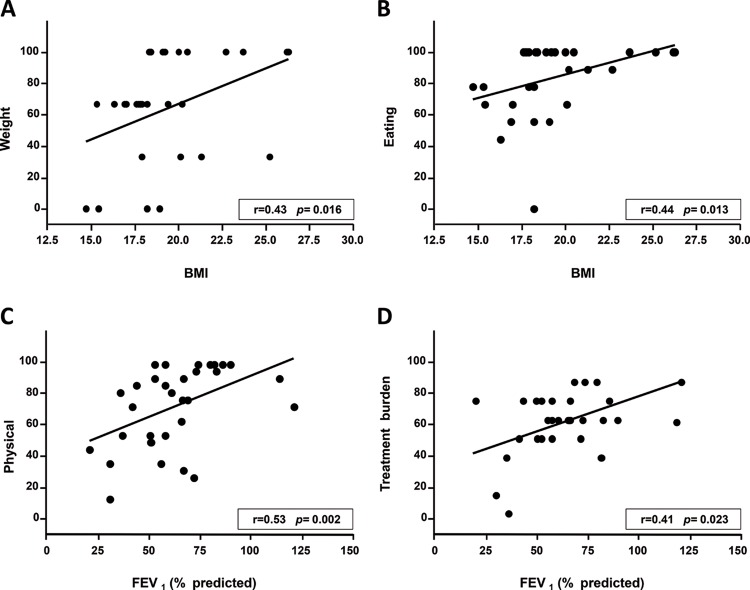
A) The correlation between the weight domain and body mass index (BMI); B) the correlation between the eating domain and BMI; C) the correlation between the physical domain and FEV_1_; and D) the correlation between the treatment burden domain and FEV_1_.

**Figure 2:**
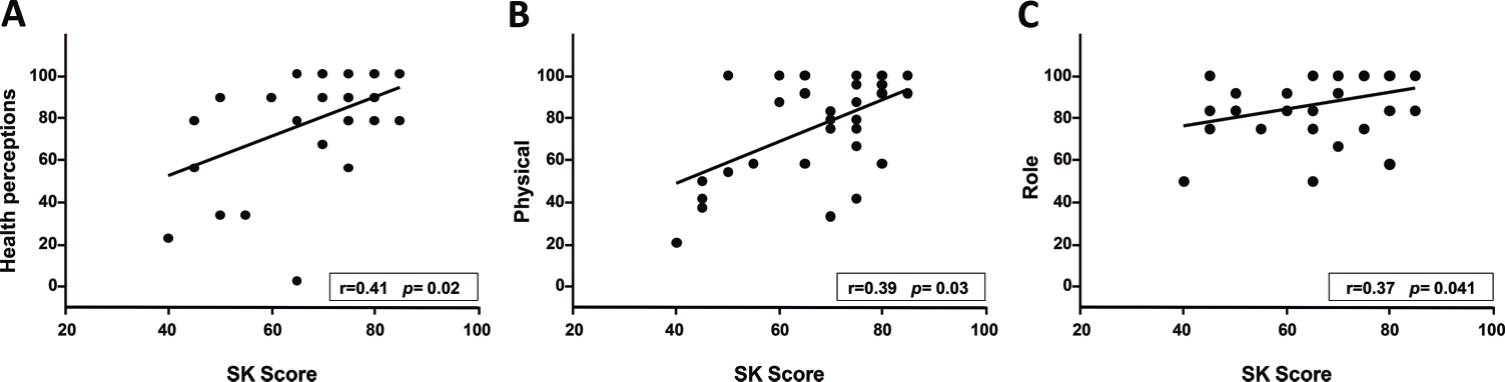
A) The correlation between the health perceptions domain and the SKS; B) the correlation between the physical functioning domain and the SKS; C) the correlation between the role domain and the SKS.

A significant correlation was found between BMI and the weight and eating domains as well as between FEV_1_ and the physical and treatment domains (Figure 1). SKS was also significantly correlated with the health, physical, and role domains (Figure 2). The remainder of the correlations tested were not significant.

**Figure 3:**
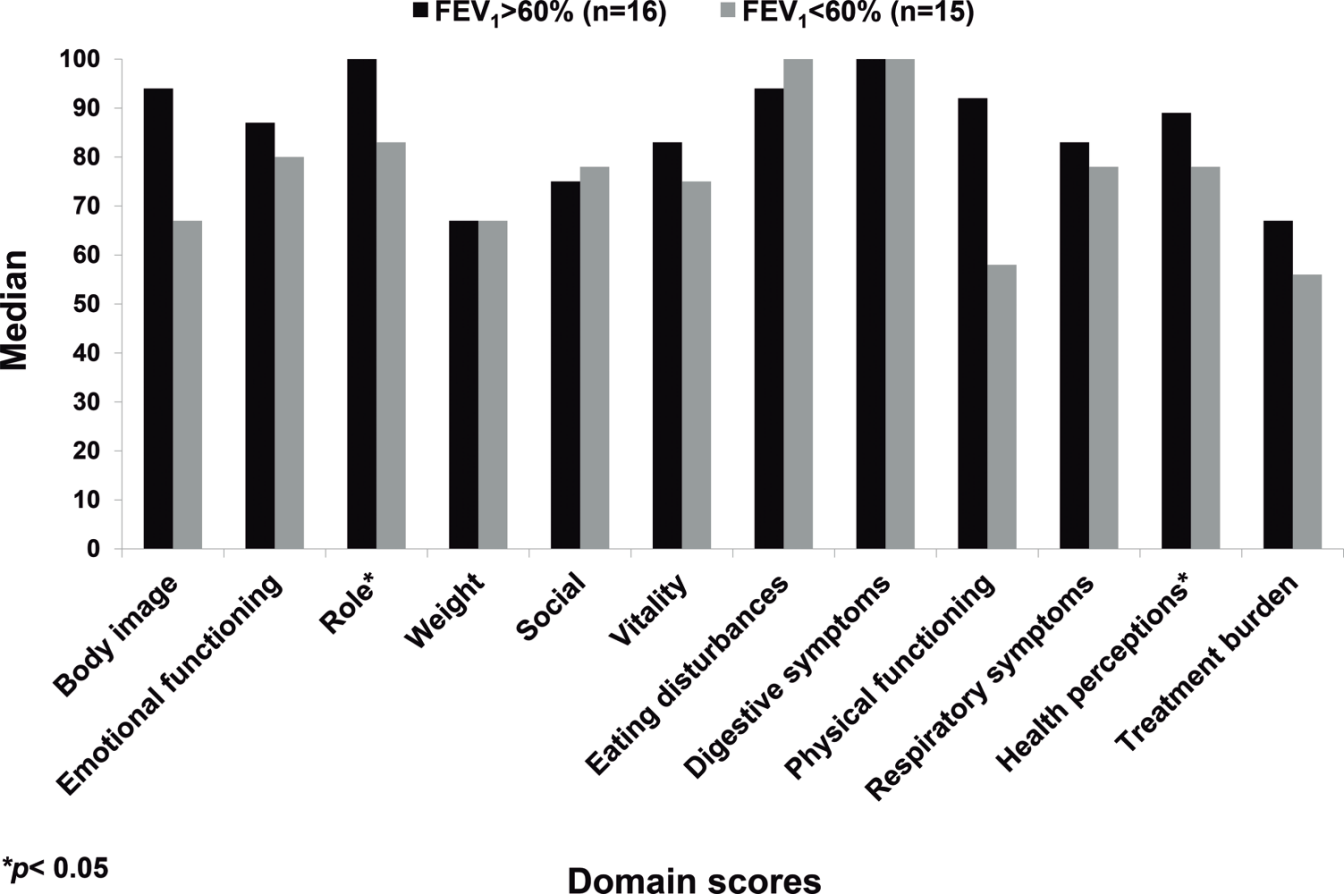
The CFQ-R domain scores for FEV_1_ values above and below 60% of the predicted value.

No CFQ-R domain exhibited a significant difference according to gender. The groups with FEV_1_ values above and below 60% of the predicted value differed with regard to the role and health domains (Figure 3).

## DISCUSSION

In the present study, the median CFQ-R domains scores ranged from 66.7 to 100, which indicates a satisfactory quality of life for most of the patients. Positive correlations were found between the patients' BMI and their scores on the weight and eating domains; between their FEV_1_ scores and the physical and treatment burden domains; and between the SKS and the physical, health, and role domains. These findings indicate the importance of evaluating patient's health perceptions as measured using the CFQ-R.

The CFQ-R domain scores observed in our sample were similar to the reference values obtained from a large US sample of patients with CF, in which 4,679 were adolescents/adults 25, as well as those of other studies 7,17.

Healthy children and adolescents who completed the same questionnaire exhibit considerable difference in their scores depending on age and gender. Their median scores varied from 67 to 100, and more than 65% of these individuals do not obtain the maximum score for most domains 13. In addition, 99.7% of healthy children and adolescents report the presence of some degree of tiredness/exhaustion as measured by the vitality domain 13. These data show that even healthy children exhibit variable CFQ-R scores, indicating that low scores on the CFQ-R domains might reflect circumstances associated with the normal development of children and adolescents and not QoL impairments related to CF itself.

In the present study, the domains with the lowest scores were weight and treatment burden (both with median of 66.7). In Quittner's et al. studies, the domains with the lowest scores in the same age range were treatment burden, weight, and vitality (medians of 64, 71, and 67, respectively) 9 as well as vitality, treatment burden, and respiratory symptoms (means of 59.5, 61.5, and 62.9, respectively) 25. The lowest scores in a study of the validation of the CFQ-R in Germany were for vitality and respiratory domains, whereas the mean score of the treatment burden domain varied from 55 to 67.9, depending on disease severity 17.

The correlations found between patient BMI and their weight and eating domains agree with the reports of other authors. The aforementioned German validation study found that well-nourished individuals exhibit higher weight, eating, and body image domain scores than those with BMIs below 19 17. A Spanish-language CFQ-R validation study found correlations between BMI and the weight domain as well as between a fat-free mass index and the physical, vitality, body image, and eating domains 16. One study assessing children and adolescents with an early diagnosis of CF through a new-born screening program found positive correlations amongst body weight, height, and BMI z-scores and the physical and body image domains. The authors observed that reaching both short-term (BMI) and long-term (height) maximum growth is beneficial to quality of life 26.

The highest scores in the present study were those associated with the digestive symptom and eating domains, with medians of 100 in each case, which is similar to previous studies 11,17,25. These data seem to contradict the data relative to the weight domain; this result might be because the CFQ-R weight domain includes one question related to difficulty in gaining weight, whereas the digestive and eating domains include three additional generic questions on topics such as abdominal pain, diarrhoea, and appetite.

The median FEV_1_ value in the present study was 61%, which corresponds to a moderate degree of obstructive lung disease. The FEV_1_ values were correlated with the CFQ-R physical and treatment burden domain scores. Other authors found correlations between FEV_1_ values and the physical, health, body image, and other domains 11,17,25,27,28.

Some longitudinal studies assessing quality of life have shown that patient-reported outcomes such as the CFQ-R and others are sensitive enough to evaluate changes in health status over time 29,30, whereas others have not 31. When the Cystic Fibrosis Quality of Life Questionnaire was applied to 234 adults with CF, positive correlations were found between FEV_1_ values and all questionnaire domains except for body image. In addition, reductions in both lung function and quality of life were found over a 12-year period 29.

Although the current study had a limited sample size, significant differences were found between patients with FEV_1_ above or below 60% of the predicted value with regard to the role and health domains. The physical and treatment domain scores tended to be higher for the group with FEV_1_ values above 60% compared with the group with those below 60% (physical: 83±21 *vs*. 67±24, respectively, *p*=0.06; treatment: 71.5±13 *vs*. 56.3±22, respectively, *p*=0.06). Other authors also observed significant differences in the CFQ-R domains according to disease severity as evaluated by lung function (FEV_1_ or FVC) or clinical score (SKS) 10,16,32.

Significant differences were not found in a Brazilian study with regard to any of the CFQ-R domains amongst patients older than 14 years with SKSs above or below 70 32. The present study did not categorise the patients by SKS; however, significant correlations were found between the SKS and the physical, health, and role domain scores.

One limitation of the present study is its small sample size, which precludes us from classifying our patients by age group. Previous studies have shown that older patients have worse scores on most of domains compared with younger patients 16,17. In healthy children, symptoms related to the digestive domain occur more frequently amongst the youngest participants, and manifestations related to body image tend to increase with age 13. Another limitation is that approximately 50% of the eligible patients did not participate, which might compromise the external validity of certain findings, reinforcing the need for more studies on this topic amongst this age group.

Concerning gender, the present study did not find differences in the CFQ-R domains, which might be related to the small sample size. Lower health-related QoL are usually reported by female adolescents with CF 33 as well as those in the general population 13 compared with their male counterparts, except for the domains related to body appearance. The reason for this result might be that girls often want to be thin, and CF helps them to achieve that goal. These gender differences in HRQoL have been associated with more severe pulmonary disease among female adolescents and adults with CF 14.

The average time required to complete the questionnaire was 15 minutes, indicating that it can be appropriately administered during routine medical visits without compromising care.

In conclusion, the scores on all of the CFQ-R domains of the patients studied, although heterogeneous, showed a minimum median of approximately 70, which indicates a relatively satisfactory level of quality of life. Some of the CFQ-R domains showed significant correlations with clinical parameters, thereby affirming that this instrument has consistent associations with clinical outcomes. The CFQ-R is easy to apply and can be answered over a brief time period. In addition to cross-sectional assessments, the CFQ-R should also be used in the clinical context or for annual evaluations and to assess therapeutic interventions.

## AUTHOR CONTRIBUTIONS

Gancz DW conceptualized and designed the study, performed all data collection, participated in all data analyses, drafted the initial manuscript, revised and approved the final manuscript as submitted. Cunha MT helped in CFQ-R data collection and analyses, drafted the initial manuscript, revised and approved the final manuscript as submitted. Leone C contributed to the study design, the statistical analysis and the interpretation of the results, revised the manuscript and approved the final manuscript as submitted. Rodrigues JC conducted the initial analyses, revised the manuscript and approved the final manuscript as submitted. Adde FV conceptualized and designed the study, performed all data analysis, drafted the initial manuscript, revised and approved the final manuscript as submitted.

## Figures and Tables

**Table 1:** Patient demographics and clinical characteristics.

**Age (years)**	
Mean±SD	16.4±2.3
Median (range)	15.7 (14.1-21)
**Gender n (%)**	
Male	20 (64.5)
Female	11 (35.5)
**Mutations n (%)**	
F508del homozygous	11 (35.5)
F508del heterozygous	11 (35.5)
Others	7 (23)
Not investigated	2 (6)
**Age at diagnosis (years)**	
Mean±SD	4.1±4.3
Median (range)	3 (0.1-15)
**Pancreatic insufficiency, n (%)**	27 (87)
**Bronchial colonisation, n (%)**	
Chronic *Pseudomonas aeruginosa*	21 (67.7)
Intermittent *Pseudomonas aeruginosa*	5 (16.1)
Intermittent/chronic *Staphylococcus aureus*	23 (74.2)
Intermittent/chronic MRSA	4 (12.9)
Intermittent/chronic *Burkholderia cepacia*	2 (6.4)
**SKS**	
Mean±SD	66.9±12.7
Median (range)	70 (40-85)
**BMI**	
Mean±SD	19.3±2.9
Median (range)	18.4 (14.7-26.3)
**Spirometry (% predicted)**	
**FVC**	
Mean±SD	76.9±21.7
Median (range)	76 (38-145)
**FEV_1_**	
Mean±SD	62.9±22.6
Median (range)	61 (21-121)
**Treatments, n (%)**	
Pancreatic enzymes	27 (87)
Fat-soluble vitamins	25 (80.6)
Inhaled dornase alfa	29 (93.5)
Inhaled tobramycin	18 (58)
Ursodeoxycholic acid	11 (35.5)
Insulin	4 (12.9)
Metformin	1 (3.2)
Home oxygen therapy	4 (12.9)

MRSA, methicillin-resistant *Staphylococcus aureus*; BMI, body mass index; FVC, forced vital capacity; FEV_1_, forced expiratory volume in one second.

**Table 2:** CFQ-R domains scores.

Domains	Mean±SD	Median	Minimum	Maximum
Body image	72.8±29	77.8	11.1	100
Emotional functioning	81±15.4	80	40	100
Role	86.8±15.2	91.7	50	100
Weight	63.5±35.9	66.7	0	100
Social	74±16.4	77.8	38.9	100
Vitality	78.5±16.8	83.3	33.3	100
Eating disturbances	83.5±23.3	100	0	100
Digestive symptoms	93.2±11.7	100	66.7	100
Physical functioning	75.4±23.7	83.3	20.8	100
Respiratory symptoms	76.3±11.4	77.8	44.4	94.4
Health perceptions	77±25.3	88.9	0	100
Treatment burden	64.1±19.4	66.7	11.1	88.9
